# Formation of β-glucogallin, the precursor of ellagic acid in strawberry and raspberry

**DOI:** 10.1093/jxb/erw036

**Published:** 2016-02-16

**Authors:** Katja Schulenburg, Antje Feller, Thomas Hoffmann, Johannes H. Schecker, Stefan Martens, Wilfried Schwab

**Affiliations:** ^1^Biotechnology of Natural Products, Technische Univeristät München, Liesel-Beckmann-Str. 1, D-85354 Freising, Germany; ^2^Department of Food Quality and Nutrition, IASMA Research and Innovation Center, Fondazione Edmund Mach (FEM), Via E. Mach 1, 38010 San Michele all’Adige, (TN), Italy

**Keywords:** Ellagic acid, ellagitannin, *Fragaria*, β-glucogallin, glucose ester, glycosyltransferase, *Rubus*.

## Abstract

Ellagic acid/ellagitannins are plant polyphenolic antioxidants that are synthesized from gallic acid and have been associated with a reduced risk of cancer and cardiovascular diseases. Here, we report the identification and characterization of five glycosyltransferases (GTs) from two genera of the *Rosaceae* family (*Fragaria* and *Rubus*; *F.×ananassa* FaGT2*, FaGT2, FaGT5, *F. vesca* FvGT2, and *R. idaeus* RiGT2) that catalyze the formation of 1-*O*-galloyl-β-d-glucopyranose (β-glucogallin) the precursor of ellagitannin biosynthesis. The enzymes showed substrate promiscuity as they formed glucose esters of a variety of (hydroxyl)benzoic and (hydroxyl)cinnamic acids. Determination of kinetic values and site-directed mutagenesis revealed amino acids that affected substrate preference and catalytic activity. Green immature strawberry fruits were identified as the main source of gallic acid, β-glucogallin, and ellagic acid in accordance with the highest *GT2* gene expression levels. Injection of isotopically labeled gallic acid into green fruits of stable transgenic antisense *FaGT2* strawberry plants clearly confirmed the *in planta* function. Our results indicate that GT2 enzymes might contribute to the production of ellagic acid/ellagitannins in strawberry and raspberry, and are useful to develop strawberry fruit with additional health benefits and for the biotechnological production of bioactive polyphenols.

## Highlight

Biochemical characterizations of glycosyltransferases in green strawberry and raspberry fruits demonstrate formation of β-glucogallin as being an initial step for biosynthesis of the polyphenolic antioxidants, ellagic acid/ellagitannins.

## Introduction

Plant phenols are mostly products of the phenylpropanoid and shikimate pathway, and comprise a large variety of compounds (Quideau *et al.*, 2011). They have been associated with miscellaneous *in planta* functions including defense against bacteria, fungi, viruses (Akiyama *et al.*, 2001; Reddy *et al.*, 2007), and animal herbivores (Barbehenn and Constabel, 2011) as well as with protection against solar radiation (Harborne and Williams, 2000). In addition, polyphenolic components of major dietary constituents have been linked to a number of potential health benefits (Quideau *et al.*, 2011). Among the bioactive polyphenols are tannins, which can be divided into condensed tannins (proanthocyanidins) and hydrolyzable tannins (ellagitannins and gallotannins). The latter are heterogeneous polymers formed from phenolic acids, especially gallic acid, esterified with simple sugar molecules (Niemetz and Gross, 2005). Recent studies revealed that ellagitannins and ellagic acid (dimeric gallic acid) can be effective remedies against various human diseases such as breast cancer, prostate cancer, and cardiovascular and neurodegenerative disorders, and they are thought to play important roles in long-term health protection (Landete, 2011; Giampieri *et al.*, 2012). Although the biological significance of these compounds is widely acknowledged, there is still little known about the genetic background of their biosynthetic pathway.

Ellagitannins and gallotannins probably derive from 1,2,3,4,6-pentagalloylglucose by addition of further galloyl residues or by oxidation, whereas the precursor is formed by successive galloylation of glucose (Niemetz and Gross, 2001). The biosynthesis starts with the formation of β-glucogallin (1-*O*-galloyl-β-d-glucopyranose), which is generated by esterification of gallic acid and glucose and gives rise to di-, tri-, tetra-, and pentagalloylglucose by transesterification reactions (Niemetz and Gross, 2005). Although formation of β-glucogallin is the essential step in ellagitannin/gallotannin biosynthesis, only four genes coding for gallic acid UDP-glucose glucosyltransferases (GTs) have been isolated from *Vitis vinifera* (Khater *et al.*, 2012) and *Quercus robur* (Mittasch *et al.*, 2014) to date. The recombinant enzymes showed promiscuous activity towards several (hydroxyl)benzoic and (hydroxyl)cinnamic acids including gallic acid that resulted in the formation of the related 1-*O*-acyl glucose esters, while UDP-glucose was acting as the activated donor substrate.

Strawberry and raspberry contain high amounts of bioactive polyphenols, in particular ellagic acid/ellagitannins (Aaby *et al.*, 2005, 2012; Vrhovsek *et al.*, 2012), being about three times higher than in other fruits or nuts (Häkkinen *et al.*, 1999; Williner *et al.*, 2003; Wang *et al.*, 2008). Thus, we employed strawberry (*Fragaria×ananassa* and *Fragaria vesca*) and raspberry (*Rubus idaeus*) as plant systems to study genes that encode GTs which are able to catalyze the formation of β-glucogallin amongst other 1-*O*-β-d-glucose esters. Comprehensive *in vitro* enzyme assays confirmed the activity of the recombinant proteins towards gallic acid. Feeding of isotopically labeled gallic acid to fruits of stable transgenic plants verified the *in planta* function of GT enzymes in *F.×ananassa*.

## Materials and methods

### Plant material

*Fragaria×ananassa* cv. Calypso and Elsanta, *F. vesca* cv. Yellow Wonder, and stable transgenic *FaGT2*-silenced (Calypso background) strawberry plants (Lunkenbein *et al.*, 2006) were cultivated in the Call Unit for plant research of the TUM School of Life Sciences in Freising, Germany. The transgenic lines were rejuvenated continuously by collecting clones from stolons, which strawberry plants regularly form. Fruits were collected from April until August 2013 and 2014, directly freeze-dried after harvest, and stored at –20 °C until processed further. *Rubus idaeus* cv. Tulameen and *F. vesca* (unknown accession) were grown on the campus of Fondazione Edmund Mach in Vigalzano di Pergine, Italy. Fruits were collected in summer 2013 (*F. vesca*) or 2014 (*R. idaeus*), directly freeze-dried after harvest, and stored at –80 °C until processed further.

### Chemicals

All chemicals, solvents, and reference compounds were obtained from Roth (Karlsruhe, Germany), Sigma-Aldrich (Steinheim, Germany), or Fluka (Steinheim, Germany), except where otherwise stated.

### Construction of expression plasmids

The full-length ORFs of FaGT2* and *FaGT5* were obtained from cDNA of ripe fruit of *F.×ananassa* cv. Elsanta by amplification with primers introducing *Bam*HI/*Not*I (FaGT2*) and *Eco*RI/*Xho*I (*FaGT5*) restriction sites (Supplementary Table S1 at *JXB* online). The genes were subcloned into the pGEM-T Easy vector (Promega, Madison, WI, USA). The sequence of *FaGT2* was synthesized by Eurofins Genomics (Ebersberg, Germany), already flanked by *Bam*HI and *Not*I restriction sites, and subcloned into the pEX-K4 vector. Subsequently, the genes were cloned into the pGEX-4T-1 vector (Amersham Bioscience, Freiburg, Germany) in-frame with the N-terminal tag. Sequencing of the complete insert (Eurofins Genomics) confirmed the identity of the cloned sequences. The full-length ORF of *FvGT2* was amplified from cDNA of turning fruits (stage 3) of *F. vesca* and cloned into pGEX4T-1 as a *Bam*HI/*Not*I fragment. The full-length ORF of *RiGT2* was amplified from cDNA of turning fruits (stage 3) of *R. idaeus* ‘Tulameen’ and cloned into pGEX4T-1 as a *Bam*HI/*Eco*RI fragment. Primer sequences to amplify *RiGT2* were obtained from Judson Ward (personal communication). All constructs were verified by DNA sequencing.

### Site-directed mutagenesis

Site-directed mutagenesis of FaGT2* was carried out according to the QuikChange^®^ protocol (Agilent Technology, Santa Clara, USA). The primers were designed as described in the manual, and the success of the mutation was confirmed by sequencing (Supplementary Table S2).

### Heterologous protein expression

The recombinant proteins were expressed in *Escherichia coli* BL21 (DE3) pLysS (Novagen, Darmstadt, Germany). Preparatory cultures were grown overnight at 37 °C in LB medium containing 100 µg ml^−1^ ampicillin and 23 µg ml^−1^ chloramphenicol. The next day, 1 liter of LB was inoculated with 10ml of the pre-culture and grown at 37 °C at 160rpm until the OD_600_ reached 0.5–0.7. To induce protein expression, 1mM isopropyl-β-d-1-thiogalactopyranoside (IPTG) was added and the cultures were kept at 16–18 °C at 160rpm overnight before they were harvested by centrifugation (10min, 5000 *g*) and stored at –80 °C.

### Cell lysis and purification

Recombinant glutathione *S*-transferase (GST) fusion proteins were purified by GST Bind resin (Novagen) following the manufacturer’s instructions. Cells were disrupted by sonication (3min, 5s intervals, 50% intensity; Bandelin Sonoplus, Berlin, Germany) after re-dissolving them in 10ml of binding buffer containing 10 µM of the proteinase inhibitor phenylmethylsulfonyl fluoride. The crude protein extract was incubated for 2h with the resin in order to bind the GST fusion proteins. The recombinant proteins were eluted with GST elution buffer containing 100mM reduced glutathione and quantified (Bradford, 1976). The identity of the heterologously expressed proteins was verified by SDS–PAGE and western blot (anti-GST antibody).

### Radiolabeled enzyme activity assay and kinetics

Substrate screens were performed at optimized conditions (100mM Tris-HCl pH 7.0, 30 °C), containing 2 µg of recombinant protein, 1mM substrate, and 10 010 pmol UDP-glucose (9980 pmol unlabeled and 30 pmol labeled UDP-[^14^C]glucose). Empty vector protein extract was processed under identical conditions and employed as the negative control. All reactions were stopped by addition of 1ml of water-saturated 1-butanol. The mixture was vortexed and the phases were separated by centrifugation (2min at 12 000 *g*). An aliquot of the organic phase (800 µl) was mixed with 2ml of Pro Flow P+ cocktail (Meridian Biotechnologies Ltd, Epsom, UK) and radioactivity was determined by liquid scintillation counting (LSC; Tri-Carb 2800TR Perkin Elmer, Waltham MA, USA). The kinetic data were determined with increasing concentrations of substrate (50 µM to 1mM) and the same fixed mixture of labeled and unlabeled UDP-glucose (10 010 pmol) which was used for the screens. Assays were stopped after 30min. To determine the kinetic data of UDP-glucose, a fixed concentration of 4-hydroxybenzoic acid (200 µM) was used and varying mixtures of unlabeled UDP-glucose and UDP-[^14^C]glucose (BIOTREND Chemikalien GmbH, Koeln, Germany) ranging from 50 µM to 1mM. The kinetic data were calculated from Hanes–Woolf plots. To evaluate the obtained constants, non-linear regression of the Michaelis–Menten equation was used. Only data that matched both calculations were included in the Results section. The GST tag was not removed prior to determination of the kinetic data. Therefore, we used the molecular weight of the fusion protein for calculation of *k*_cat_.

### Injection of deuterium-labeled gallic acid into fruits of transgenic *FaGT2*-silenced strawberry plants

The injection conditions were optimized prior to the *in vivo* experiment (Supplementary Fig. S1). 3,4,5-Trihydroxybenzoic-2,6-d_2_ acid (10mM) was injected into small green fruits of stable transgenic *FaGT2*-silenced plants (Lunkenbein *et al.*, 2006) and *F.×ananassa* cv. Calypso control plants. After a 24h incubation, the fruits were harvested, freeze-dried, and stored at –20 °C.

### Sample extraction

For quantification of secondary metabolites in strawberry fruit, samples were extracted according to Ring *et al.* (2013). Each tissue sample was ground individually using a mortar and pestle and extracted with methanol. The extracts were evaporated to dryness in a vacuum concentrator and re-dissolved in 50 µl of water. After 1min of vortexing, 10min of sonication, and 10min of centrifugation at 16 000 *g*, the clear supernatant was used for LC-MS analysis.

### LC-MS analysis

The levels of gallic acid, β-glucogallin, and ellagic acid were determined in two tissue types (achenes and receptacles) and five ripening stages (small green, big green, white, turning, and ripe) of *F.×ananassa* cv. Calypso fruits and three ripening stages (small green, white, and ripe) of *F. vesca* cv. Yellow Wonder. It was not possible to distinguish the big green stage from the small green stage and turning from ripe fruit of the Yellow Wonder genotype, because this genotype produces small fruits and turns from green to white ripe fruits very quickly. The ripe fruits do not turn red in color. On account of this, we harvested three ripening stages and separated achenes from the pulp. The three metabolites were identified and quantified by LC-MS analysis in the positive and negative MS mode by the internal standard method (Ring *et al.*, 2013). The values were expressed as per mil (‰) equivalent of the dry weight. The identity of the metabolites was confirmed by reference compounds run under identical conditions. Levels of labeled and unlabeled compounds were determined with an Agilent 1100 HPLC/UV system (Agilent Technologies) equipped with a reverse-phase column [Luna 3u C18(2) 100A, 150×2mm; Phenomenex] and connected to a Bruker esquire3000plus ion-trap mass spectrometer (Bruker Daltonics). The system was adjusted, analysis was performed, and metabolite levels were measured according to Ring *et al.* (2013). As reference compounds, chemically pure gallic acid, β-glucogallin (LC Scientific Inc., Concord, Ontario, Canada), and ellagic acid were purchased and run under identical conditions in order to identify their retention times and mass spectra (MS and tandem MS). Gallic acid 4-*O*-glucoside was identified according to the literature (Schuster and Herrmann, 1985; Lu and Yeap Foo, 1999; Pawlowska *et al.*, 2006).

### Transcriptome data set of *F. vesca* cv. Yellow Wonder tissues

The expression of FaGT2 (AY663785; gene26265) and FaGT5 (DQ289586; gene26249) corresponding transcripts in three stages of green fruit development of *F. vesca* cv. Yellow Wonder was evaluated in a transcriptome data set publicly available at the SGR database (Darwish *et al.*, 2013; Kang *et al.*, 2013; http://bioinformatics.towson.edu/strawberry/newpage/Compare_Samples.aspx, accessed August 2015).

## Results

### Selection of putative gallic acid UDP-glucose glucosyltransferases from *F. vesca*, *F.×ananassa*, and *R. idaeus*

**Fig. 1,:**
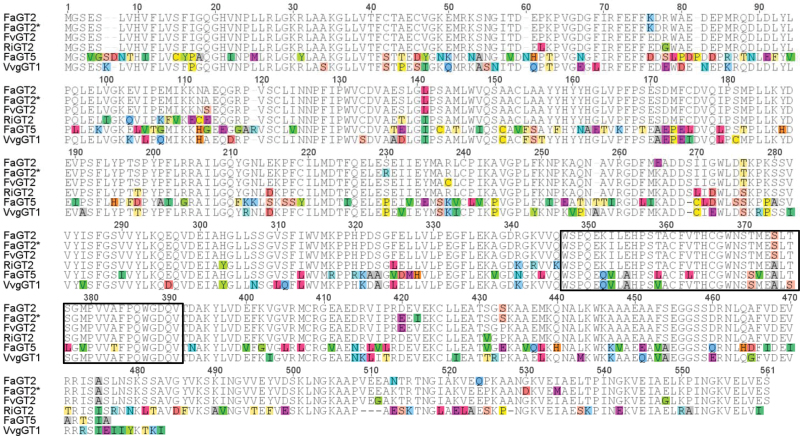
Protein sequence alignment of glucosyltransferases which catalyze the formation of β-glucogallin: garden strawberry (*Fragaria×ananassa* cv. Elsanta) FaGT2*, FaGT2, and FaGT5; woodland strawberry (*Fragaria vesca*) FvGT2; raspberry (*Rubus idaeus* cv. Tulameen) RiGT2, and grape vine (*Vitis vinifera* cv. Maccabeu) VvgGT1 (gi|363805185|gb|JN164679). This alignment was processed by ClustalX of the GENEIOUS Pro 5.5.6 program with its default parameters (Biomatters; http://www.geneious.com/, accessed August 2015). The Plant Secondary Product Glycosyltransferase (PSPG) box is boxed.

Sequence alignment and a phylogenetic comparison of three UDP-glucose:gallic acid GTs from *Vitis vinifera* (VvgGT1–VvgGT3; Khater *et al.*, 2012) with putative GTs encoded by the *F. vesca* genome (Shulaev *et al.*, 2011) yielded two sequences whose closest homologs in *F.×ananassa* have been named *FaGT2* and *FaGT5* (Supplementary Fig. S2). Recently, FaGT2 has been shown to be involved in the formation of cinnamoyl and 4-coumaroyl glucose ester in *F. × ananassa* (Lunkenbein *et al.*, 2006; Landmann *et al.*, 2007). Re-amplification of the ORF of *FaGT2* from cDNA obtained from *F.×ananassa* cv. Elsanta produced a sequence FaGT2* that differed from the published version in 18 single nucleotide polymorphisms (SNPs). These SNPs resulted in eight amino acid changes and were located before and after the Plant Secondary Product Glycosyltransferase (PSPG) box, a conserved region assumed to be responsible for the interaction with the sugar donor (Fig. 1). In addition to both FaGT2 sequences and FaGT5, we selected GT2 orthologs from *F. vesca* (FvGT2) and *R. ideaus* (RiGT2) for further analysis. Two mutants of FaGT2* were also generated by site-directed mutagenesis to test the effect of the amino acid changes between FaGT2* and FaGT2 on the catalytic activity and substrate specificity of the enzymes. VvgGT1 was used as a positive control (Khater *et al.*, 2012). The GT2 protein sequences and VvgGT1 show a pairwise identity of >80% (Fig. 1), while GT2 homologs from strawberry and raspberry even have an identity of >93%. The genes were cloned into the expression vector pGEX-4T-1. The recombinant proteins were expressed with an N-terminal GST tag and enriched by affinity purification. The presence of the enzymes was verified by SDS–PAGE and western blot.

### Enzymatic activity of GT2 homologs

A set of (hydroxy)benzoic acid and (hydroxy)cinnamic acid derivatives was selected to elucidate the substrate specificity of the GT2 homologous enzymes *in vitro*. The substrates gallic acid, 4-hydroxybenzoic acid, and protocatechuic acid were chosen because they are known precursors of hydrolyzable tannins (Haslam and Cai, 1994), while others show structural similarity (salicylic acid and gentisic acid) or their *O*-acyl glucoside has been reported in strawberry, raspberry, and grape (e.g. cinnamic acid and *p*-coumaric acid). All recombinant proteins converted gallic acid to the corresponding 1-*O*-acyl glucose ester (Table 1). FaGT2* preferred 4- and, 3-hydroxybenzoic acid and vanillic acid, but also efficiently (>90% relative activity) converted protocatechuic acid and *m*-coumaric acid. FaGT2 showed a preference for the benzoic acid derivatives as well, and esterified veratric acid with the highest efficiency. The enzyme activity of FvGT2 and RiGT2 indicated a higher affinity for cinnamic acid derivatives, and both enzymes glucosylated benzoic acid derivatives likewise with a comparatively high activity. With the exception of veratric acid, FaGT5 clearly favored hydroxycinnamic acid derivatives (sinapic acid 100%). VvgGT1 preferred *m*-coumaric acid but also converted veratric acid and sinapic acid with a high efficiency. All enzymes were able to use sorbic acid as the acceptor molecule and, except for FaGT2, showed a low activity towards salicylic acid and gentisic acid. The formation of ester bonds was confirmed by alkaline hydrolysis and visualized by LC-MS, exemplarily shown for sinapoyl glucose ester (Supplementary Fig. S3).

**Table 1.** Relative enzymatic activities of the investigated GTs towards a set of acceptor substrates

Substrates	FaGT2*	FaGT2	FvGT2	RiGT2	FaGT5	VvgGT1
Sorbic acid	32	66	45	81	13	93
Salicylic acid	0	88	1	4	1	6
3-Hydroxbenzoic acid	99	95	80	77	3	39
4-Hydroxybenzoic acid	100	89	77	89	15	69
Protocatechuic acid	92	95	71	68	6	61
Gentisic acid	0	81	5	15	1	13
**Gallic acid**	**34**	**89**	**51**	**43**	**5**	**42**
Vanillic acid	99	98	83	78	21	69
Veratric acid	76	100	82	64	92	94
Syringic acid	87	94	75	59	13	86
Cinnamic acid	38	80	57	93	14	31
*o*-Coumaric acid	76	87	82	99	11	82
*m*-Coumaric acid	95	63	100	90	21	100
*p*-Coumaric acid	53	75	95	100	48	90
Caffeic acid	65	81	92	91	17	80
Ferulic acid	50	5	61	65	36	61
3,4-Dimethoxycinnamic acid	85	55	88	75	34	75
Sinapic acid	89	17	93	78	100	99

The relative enzymatic activities (%) were determined by radiochemical analysis with UDP-[^14^C]glucose and refer to the highest level of extractable radioactivity that was measured for every enzyme. FaGT2* 100% ≙ 1.0 nkat mg^−1^; FaGT2 100% ≙ 1.4 nkat mg^−1^; FvGT2 100% ≙ 1.3 nkat mg^−1^; RiGT2 100% ≙ 1.9 nkat mg^−1^; FaGT5 100% ≙ 2.5 nkat mg^−1^; VvgGT1 100% ≙ 1.4 nkat mg^−1^. Empty vector control was always <1% and was subtracted from the relative concentrations.

### Biochemical characterization

To determine the kinetic values, assays were performed with optimized conditions (100mM Tris-HCl buffer, pH 7, 2 µg of purified enzyme, 30 °C). For biochemical characterization of UDP-glucose, a fixed *p*-hydroxybenzoic acid concentration (200 µM) was used. The kinetic parameters were determined from Hanes–Woolf plots and non-linear regression of the Michaelis–Menten equation. The results confirm the high activity of the tested enzymes towards gallic acid, except for FaGT5 (Table 2). FaGT2*, FaGT2, FvGT2, RiGT2, and VvgGT1 show a higher substrate affinity (lower *K*_M_) and enzyme efficiency (*k*_cat_/*K*_M_) towards almost all of the tested substrates including gallic acid than FaGT5. FaGT5 preferred sinapic acid, as the *k*_cat_/*K*_M_ value of 15555s^−1^ M^−1^ for this acid exceeded the corresponding values of the other substrates tested.

**Table 2.** Kinetic data of FaGT2*, FaGT2, FvGT2, RiGT2, FaGT5, and VvgGT1 determined for the substrates gallic acid, 4-hydroxybenzoic acid, vanillic acid, syringic acid, cinnamic acid, sinapic acid, and UDP-glucose

Enzyme	Substrate	*K*_M_ (µM)	*k*_cat_ (s^−1^)	*k*_cat_/*K*_M_ (s^−1^ M^−1^)
FaGT2*	**Gallic acid**	**96±3.6**	**0.8±0.04**	**8333**
	4-Hydroxybenzoic acid	315±8.4	2.0±0.06	6349
	Vanillic acid	364±12.8	2.1±0.04	5769
	Syringic acid	262±7.0	1.8±0.07	6870
	Sinapic acid	35±3.0	1.2±0.04	34285
	UDP-glucose	378±2.3	3.8±0.07	10052
FaGT2	**Gallic acid**	**264±9.9**	**1.8±0.01**	**6818**
	4-Hydroxybenzoic acid	899±12.3	2.2±0.17	2447
	Vanillic acid	621±12.5	2.1±0.04	3381
	Syringic acid	874±6.2	2.6±0.1	2974
	Sinapic acid	83±4.8	2.5±0.17	30120
	UDP-glucose	492±5.0	4.9±0.15	9959
FvGT2	**Gallic acid**	**232±8.3**	**1.1±0.05**	**4741**
	UDP-glucose	318±11.9	2.2±0.18	6918
RiGT2	**Gallic acid**	**80±8.1**	**1.6±0.05**	**20000**
	UDP-glucose	417±16.7	6.6±0.07	15827
FaGT5	4-Hydroxybenzoic acid	515±6.5	0.5±0.04	9708
	Vanillic acid	94±1.5	0.9±0.02	9574
	Syringic acid	518±10.0	0.5±0.07	9652
	Sinapic acid	45±1.1	0.7±0.02	15555
	UDP-glucose	529±3.0	4.5±0.23	8506
VvgGT1	**Gallic acid**	**72±3.5**	**1.1±0.03**	**15277**
	4-Hydroxybenzoic acid	73±2.0	2.0±0.07	27397
	Vanillic acid	60±4.7	1.8±0.10	30000
	Syringic acid	155±2.0	2.0±0.01	12903
	Sinapic acid	52±2.5	1.9±0.12	36538
	UDP-glucose	439±16.9	10.1±0.23	22779

### Site-directed mutagenesis of FaGT2*

**Fig. 2.**
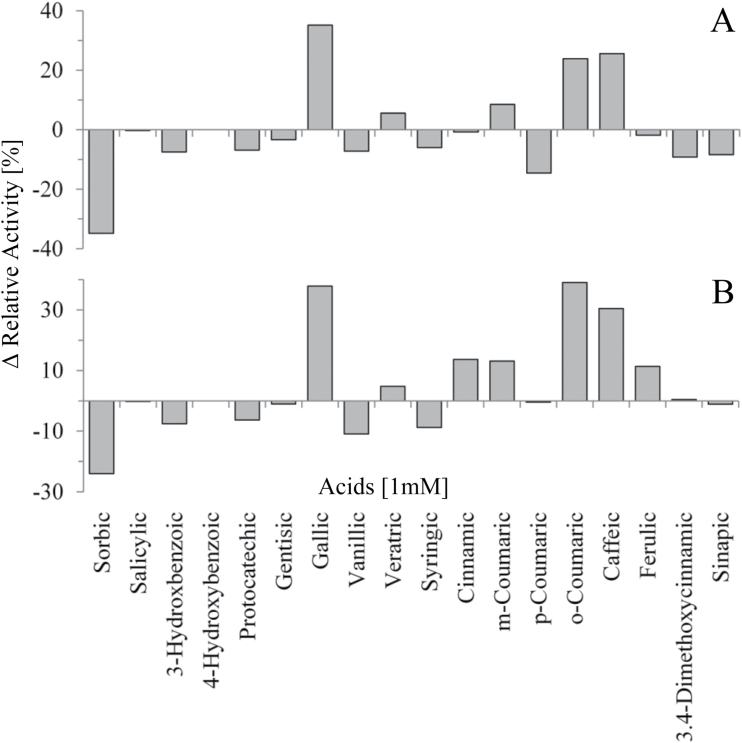
The difference (Δ) of the relative enzymatic activities of the mutants FaGT2*_R230S (A) and FaGT2*_E420D_I422V (B) towards different substrates was calculated against the respective activities of FaGT2*.

FaGT2* and FaGT2 show a pairwise identity of >98%. Eighteen SNPs result in eight amino acid changes. To test the effects of the amino exchange on the substrate specificity, three amino acids were reversed in FaGT2* by site-directed mutagenesis. Because no amino acid change was observed in the PSPG box, the presumed region interacting with the UDP-sugar, we decided to mutate amino acids in the vicinity of the PSPG box (E420 and I422) and an amino acid (R230) close to a region which was identified by Bönisch *et al.* (2014*a*) to be in the vicinity of the active site. In mutant FaGT2*_R230S, an arginine at position 230 was mutated into a serine while in mutant FaGT2*_E420D_I422V two amino acids, glutamate and isoleucine, were changed to aspartate and valine, respectively. A substrate screening was performed with 18 acids, and the difference (Δ) in the relative activities of the mutants towards the substrates was calculated against the relative activities of FaGT2* (Fig. 2). Both mutants showed an enhanced enzymatic activity towards gallic acid (35% and 38% for FaGT2*_R230S and FaGT2*_E420D_I422V, respectively), *o*-coumaric (24% and 39%), and caffeic acid (25% and 31%), whereas formation of sorbic acid glucose ester was decreased (35% and 24%).

### Metabolite analysis

**Fig. 3.**
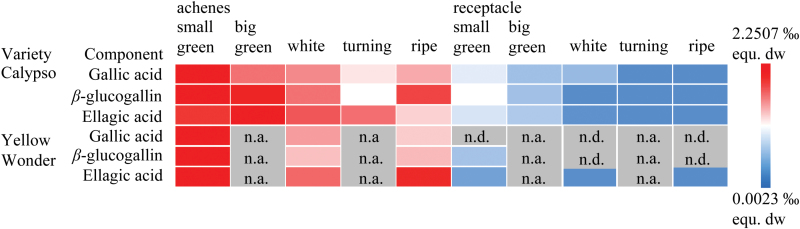
Heatmap of the relative concentration of gallic acid, β-glucogallin, and ellagic acid in strawberry tissues of *F.×ananassa* cv. Calypso and *F. vesca* cv. Yellow Wonder. Metabolite levels were determined by LC-MS and are visualized by color code (right). Mean and SD in per mil equivalents of the dry weight (‰ equ. dw) are shown in Supplementary Table S1. Achenes were removed from receptacles post-harvest and analyzed separately. n.a., not analyzed; n.d., not detectable, *n*=3–5 biological replicates.

In *F.×ananassa* cv. Calypso, the precursor molecules gallic acid and β-glucogallin and the final product of ellagitannin biosynthesis, ellagic acid, are strongly enriched in achenes of the green stage (Fig. 3). The levels decreased towards the late stages of ripening. Similar trends were observed for the levels of the ellagitannin precursors in receptacles, as well as for the *F. vesca* cv. Yellow Wonder tissues. Overall, the level of analyzed metabolites was higher in achenes than in receptacles and higher in *F.*×*ananassa* cv. Calypso than in fruits of *F. vesca* cv. Yellow Wonder (Supplementary Table S3). Thus, in the Calypso and Yellow Wonder genotypes, the investigated metabolites of ellagitannin biosynthesis are more abundant in the early stages of fruit development than in ripe fruits.

### Confirmation of FaGT2*/FaGT2 activity *in vivo*

**Fig. 4.**
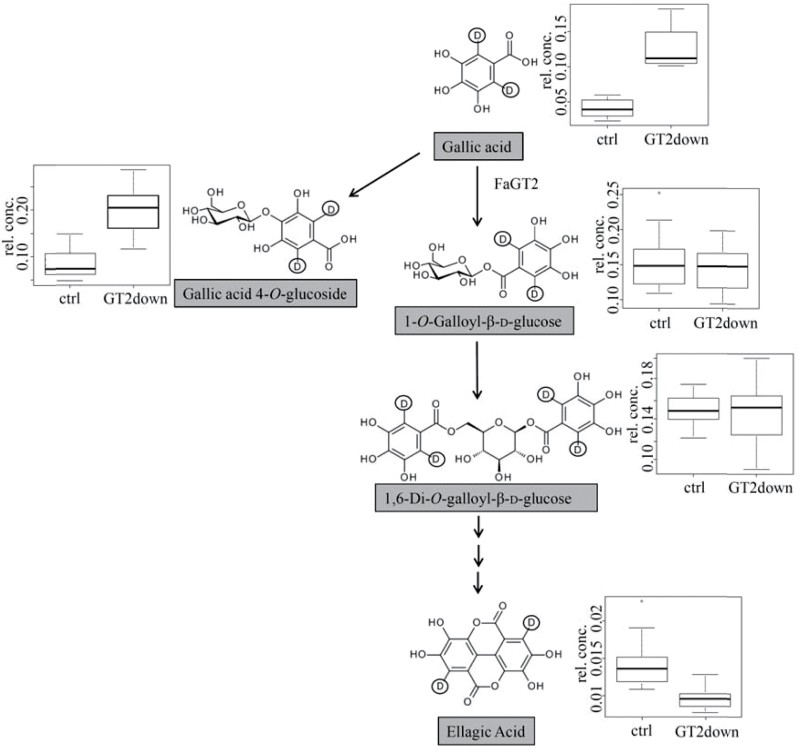
Proposed ellagic acid biosynthesis pathway with putative molecular structure of precursors. Deuterium residues are highlighted by a circle. Box plots show the relative concentration in per mil equivalent of the dry weight of the labeled metabolites in control fruit (ctrl) and in *FaGT2* silenced fruit (GT2down).

To confirm that FaGT2*/FaGT2 act as a gallic acid GT *in vivo*, aqueous solutions of deuterium-labeled gallic acid were injected into both *F.×ananassa* cv. Calypso control fruits and *FaGT2*-silenced fruits of stable transgenic strawberry plants. The generation of the stable antisense transgenic *FaGT2i* line has been described by Lunkenbein *et al.* (2006). After 24h, the levels of d_2_-gallic acid, and the downstream metabolites d_2_-β-glucogallin, d_2_-gallic acid glucoside, d_2_-di-galloyl glucose, and d_2_-ellagic acid were quantified by LC-MS (Fig. 4). A higher level of d_2_-gallic acid and d_2_-gallic acid 4-*O*-glucoside and a lower level of d_2_-ellagic acid were detected in the transgenic fruits compared with control fruits. The 4-*O*-glucoside was unambiguously identified by LC-MS in comparison with authentic reference material (Supplementary Fig. S4). Since the pathway to β-glucogallin is severely blocked, the surplus gallic acid probably results in a shift of the downstream metabolism in favor of formation of gallic acid 4-*O*-glucoside. In contrast, the amount of d_2_-β-glucogallin and d_2_-di-galloyl glucose remained unchanged.

## Discussion

### Putative gallic acid UDP-glucose GTs in *F. vesca*, *F.×ananassa*, and *R. idaeus*

GTs comprise a diverse class of enzymes that transfer activated sugar compounds onto a large number of acceptor molecules, including secondary metabolites, proteins, lipids, and other sugars (Yonekura-Sakakibara and Hanada, 2011). The classification of this enzyme class is based on the catalytic mechanism and on sequence and structure homologies (Campbell *et al.*, 1997; Coutinho *et al.*, 2003). FaGT2*, FaGT2, FvGT2, RiGT2, and FaGT5 were selected as candidates as they are close homologs to VvgGT1–VvgGT3 (Fig. 1), three gallic acid GTs from *V. vinifera.* They belong to the family 1 GTs, also known as UDP-sugar-dependent GTs, that often show specificity towards the donor molecule, mostly UDP-glucose, but they also glycosylate diverse small molecules (http://www.cazy.org/,accessed August 2015; Jones *et al.*, 2003; Sawada *et al.*, 2005). GTs cluster into phylogenetic groups, and it has been shown that within these groups proteins can have comparable substrate specificity (Radominska-Pandya *et al.*, 1999; Ross *et al.*, 2001; Gachon *et al.*, 2005). The phylogenetically related GTs investigated in this study produce 1-*O*-acyl-esters of (hydroxy)benzoic and (hydroxy)cinnamic acids and share a pairwise identity of >80% with VvgGT1 from *V. vinifera*. The GT2 homologous proteins show >90% similarity. FaGT2* and FaGT2 differ in only eight amino acids. These two sequences are most probably alleles with similar activity and arise from the octaploid genome of the garden strawberry (Pauli and Kutchan, 1998; Bönisch *et al.*, 2014*a*, *b*). Unlike FaGT5 and VvgGT1, the GT2 proteins from strawberry and raspberry have a C-terminal overhang of ~80 amino acids (Fig. 1). Similarly, UGT84A13, a hydroxybenzoic acid GT from *Q. robur*, shows 510 amino acids in total (Mittasch *et al.*, 2014). The function of the additional sequence remains unknown.

### Enzymatic activity and biochemical characterization

VvgGT1 from *V. vinifera* was employed as a positive control in the substrate screens and the kinetic assays because among the three published VvgGT enzymes, it exhibited the highest efficiency towards gallic acid (Khater *et al.*, 2012). All tested enzymes showed a broad substrate tolerance *in vitro*. FaGT2 has already been reported to glucosylate a multitude of natural and xenobiotic compounds (Landmann *et al.*, 2007). Similarly, a bi-functional resveratrol/hydroxycinnamic acid GT of Concord grape (*Vitis labrusca*) showed 99% sequence identity with VvgGT2 (corresponds to an exchange of one amino acid) and glycosylated (hydroxyl)cinnamic acids and the stilbene resveratrol (Hall and Luca, 2007). The overall acceptor substrate preference of GT2 proteins and VvgGT1 was similar, except for salicylic acid and gentisic acid, while FaGT5 showed a clear bias for (hydroxyl)cinnamic acids. Kinetic data revealed gallic acid, sinapic acid, and syringic acid as the favored substrates for the GT2 enzymes, suggesting an overall preference for tri-functionalized acceptors with functional groups in the *o*-, *m*-, and *p*-position. The *K*_M_ and *k*_cat_ values for gallic acid of all GTs studied showed a narrow range from 72 µM to 264 µM and from 0.8s^−1^ to 1.8s^−1^, respectively. The highest specificity constants *k*_cat_/*K*_M_ of 20 000s^−1^ M^−1^ and 15 277s^−1^ M^−1^ for gallic acid were calculated for RiGT2 and VvgGT1, respectively. These numbers slightly exceed the values published for VvgGT1–VvgGT3 (4231, 3194, and 3873s^−1^ M^−1^, respectively; Khater *et al.*, 2012) but are clearly lower than the value of the gallic acid GT UGT84A13 from *Q. robur* (25 952s^−1^ M^−1^; Mittasch *et al.*, 2014). In contrast, biochemical characterization confirmed that FaGT5 favored sinapic acid as the phenolic acid substrate. Strawberry fruit (*F.×ananassa*) has been identified as a rich source of sinapic acid (450 µg g^−1^ DW; Russell *et al.*, 2009) and its hexose derivatives were found to accumulate during the mid and late stage of receptacle development (Fait *et al.*, 2008) where they might contribute to monolignol biosynthesis and lignin formation in the fruit vasculature (Humphreys and Chapple, 2002).

Site-directed mutagenesis of three FaGT2* positions showed that R230S and E420D_I422V exchanges enhanced the activity of the GT towards gallic acid, *o*-coumaric acid, and caffeic acid (Fig. 2). The amino acids are not directly part of the PSPG box, the proposed active site of GTs (Wang, 2009). Nevertheless they have a notable effect on the activity and selectivity of FaGT2*. This demonstrates that amino acid residues outside of the active cleft might also cause a change in substrate specificity, as has been shown for a monoterpenol GT from *V. vinifera* (Bönisch *et al.*, 2014b).

### Metabolite analysis

The strawberry fruit is a rich source of ellagitannins and consists of two principle tissue types, pulp (receptacle) and achenes (seeds), in which these compounds are variably distributed. During the ripening process of the fruit, there seems to be an organ- and development-specific, dynamic fluctuation of the ellagitannin level (Fait *et al.*, 2008; Zhang *et al.*, 2011). In one study, β-glucogallin was mainly detected in the receptacle while higher galloylated forms accumulate in achenes (Fait *et al.*, 2008). In contrast, in a second study, similar to our result, higher levels of ellagic acid, galloyl-glucoses, and ellagitannins were found in achenes and not in the receptacle (Aaby *et al.*, 2005). The concentration of β-glucogallin peaked in the early ripening stages in achenes and the receptacle, and decreased towards the ripe stage of the fruit (Fig. 3). Gallic acid and ellagic acid levels followed a similar trend. In general, the white-fruited *F. vesca* cv. Yellow Wonder contained less tannins than red-fruited *F.×ananassa* cv. Calypso.

Gallic acid is the precursor compound of the biosynthesis of hydrolyzable tannins whereas ellagic acid is released from ellagitannins by hydrolysis at the end of the biosynthetic pathway (Niehaus and Gross, 1997). Tannins are part of the plant defense mechanism and are released from plant cells upon attack by fungi, bacteria, and insects (Silva *et al.*, 1997). They inhibit the growth of microorganisms and even offer protection against ruminants through formation of complexes with animal proteins that create an unpleasant sensation (Edelmann and Lendl, 2002). For strawberry plants, it might be beneficial to accumulate astringent and antimicrobial compounds during the generative phase to prevent the regenerative organ from infestation. In the late phase of fruit ripening, the fruit firmness decreases concomitant with an increase of the sugar content. Similarly, the reduction of the tannin level and thus the astringency contributes to the appeal of the fruit.

### Correlation of expression pattern of putative gallic acid UDP-glucose GTs and metabolite levels

A transcriptome data set of *F. vesca* cv. Yellow Wonder tissues of early developmental stages has been reported recently (Darwish *et al.*, 2013; Kang *et al.*, 2013). Transcripts corresponding to *FvGT2* are abundant in tissues of the receptacle (cortex and pith) and achenes (embryo, ghost, and wall) of small green fruit, whereas the levels decreased towards the big green stage (stage 5; Supplementary Fig. S5) consistent with the reduced levels of β-glucogallin in later stages of development (Fig. 3). Expression of FvGT2 is 20- to 30-fold higher in the wall of achenes compared with receptacle tissue (Supplementary Fig. S5) and thus correlates with our metabolite data, where small green achenes were identified as the main source of gallic acid, β-glucogallin, and ellagic acid. In contrast, the levels of *FaGT5* transcripts almost always peaked in big green fruit (Supplementary Fig. S5). Quantitative real-time PCR analysis also indicated that *FaGT2* transcripts accumulate to high levels in red, ripe strawberry fruit (Lunkenbein *et al.*, 2006). Thus, it appears that in *F.×ananassa* FaGT2 has a dual function due to the spatio-temporal expression pattern. In green fruit, FaGT2 seems to be involved in the formation of the ellagic acid precursor β-glucogallin and, in ripe fruit, concomitant with the reduction of ellagic acid/ellagitannins it might glucosylate cinnamic acid (Lunkenbein *et al.*, 2006). GTs form a vast and diverse enzyme class that comprise proteins with not only specific but also promiscuous *in planta* functions (Hall and De Luca, 2007). Enzyme promiscuity might derive from evolutionary adaption to changing environmental conditions, and proteins with a broad substrate spectrum are believed to be more evolved than single-substrate enzymes (Ulusu, 2015). Our results provide evidence that GT2 homologous proteins catalyze the formation of the first known precursor of ellagic acid biosynthesis not only in garden strawberry (*F.×ananassa*), but also in woodland strawberry (*F. vesca*) and raspberry (*R. idaeus*).

### Injection of labeled gallic acid suggests FaGT2 *in vivo* activity

Formation of ellagitannin precursors was quantified in *F.×ananassa* cv. Calypso control fruits and fruits of a stable transgenic *FaGT2* antisense line after feeding with deuterium-labeled gallic acid (Fig. 4). Fruits of the green developmental stage were selected for injection as FaGT2 expression and levels of gallic acid derivatives peaked at this stage. d_2_-gallic acid accumulated in the transgenic fruits probably due to reduced FaGT2 activity, whereas ellagic acid formation seemed to be inhibited but was not completely blocked, presumably due to 34% residual *FaGT2* expression in the transgenic fruits (Lunkenbein *et al.*, 2006). Alternatively, FaGT5 might contribute to the formation of β-glucogallin and successive products, but with significantly reduced efficiency (Table 1). In contrast, the levels of d_2_-β-glucogallin and d_2_-di-galloyl-glucose were not significantly different in control fruits when compared with levels in the transgenics. It appears that the residual enzymatic activity of FaGT2 is sufficient to maintain a constant level of the intermediates but cannot sustain the flux through the ellagic biosynthetic pathway. A new metabolite of gallic acid accumulated to high levels and was subsequently identified as gallic acid 4-*O*-glucoside (Schuster and Herrmann, 1985). Thus, the surplus gallic acid resulted in a shift of the downstream metabolism in favor of glucoside production because the pathway to ester formation is severely blocked. Clearly, this result confirms one of the *in planta* functions of the FaGT2 enzyme as gallic acid GT in immature strawberry fruit.

## Supplementary data

Supplementary data are available at *JXB* online.

Table S1. Primers used for cloning.

Table S2. Primers used for site-directed mutagenesis of FaGT2*.

Table S3. Relative concentration of gallic acid, β-glucogallin, and ellagic acid in different strawberry tissues.

Figure S1. Relative concentration of d_2_-β-glucogallin after injection of different amounts of labeled gallic acid into small green strawberry fruits.

Figure S2. Amino acid sequence alignment and phylogenetic tree of strawberry (*F. vesca*) proteins.

Figure S3. Confirmation of the formation of ester bonds by FaGT2*.

Figure S4. LC-MS analysis of β-glucogallin and gallic acid 4-*O*-glucoside.

Figure S5. Expression of FaGT2 and FaGT5 corresponding transcripts in three stages of green fruit development of *F. vesca* cv. Yellow Wonder.

Supplementary Data
