# Supplementary material for: Formation of β-glucogallin, the precursor of ellagic acid in strawberry and raspberry
Source: J Exp Bot. 2016 Feb 16;67(8):2299–308. doi: 10.1093/jxb/erw036 (PMC4809288; doi:10.1093/jxb/erw036)
Supplement: Supplementary Data [file supp_67_8_2299__index.html]

Formation of β-glucogallin, the precursor of ellagic acid in strawberry and raspberry — Formation of β-glucogallin, the precursor of ellagic acid in strawberry and raspberry — Supplementary Data 

# Formation of β-glucogallin, the precursor of ellagic acid in strawberry and raspberry

## Supplementary Data

Data files

- supplementary\_table\_S1\_S3\_figures\_S1\_S5.pdf - Supplementary Data
