# Supplementary material for: Formation of β-glucogallin, the precursor of ellagic acid in strawberry and raspberry
Source: J Exp Bot. 2016 Feb 16;67(8):2299–308. doi: 10.1093/jxb/erw036 (PMC4809288; doi:10.1093/jxb/erw036)
Supplement: Supplementary Data [file supp_erw036_supplementary_table_S1_S3_figures_S1_S5.pdf]

Supplementary Data

Formation of *β*-Glucogallin, the Precursor of Ellagic Acid in Strawberry and Raspberry

Katja Schulenburg[1], Antje Feller[2], Thomas Hoffmann[1], Johannes H. Schecker[1], Stefan Martens[2], Wilfried Schwab[1]

[1] Biotechnology of Natural Products, Technische Univeristät München, Liesel-Beckmann-Str. 1, 85354 Freising, Germany

[2] Department of Food Quality and Nutrition, IASMA Research and Innovation Center, Fondazione Edmund Mach (FEM), Via E. Mach 1, 38010 San Michele all'Adige, (TN), Italy

Table S1: Primers used for cloning

gene	nomenclature	sequence
FaGT2*	BamHI_fw	CGCGGATCCATGGGTTCCGAATCATTGGTT
	NotI_rev	ATAGTTTAGCGGCCGCTTACGACTCGACTAGTTCAAC
FaGT5	EcoRI_fw	AAAGAATTCATGGGTTCTGTGGGATCTG
	XhoI_rev	AAACTCGAGAGCAATAGAAGTCCTAGCAATCTC
FvGT2	BamHI_fw	TAAGCAGGATCCATGGGTTCCGAATCATTGGTT
	NotI_rev	CTTAGCGGCCGCTTACGATTCGACTAGTTCAACC
RiGT2	BamHI_fw	TAAGCAGGATCCATGGGTTCCGAATCATTGGTC
	EcoRI_rev	TGCTTAGAATTCTCAAATAACCAGTTCAACCTTTCC

Table S2: Primers used for site directed mutagenesis of FaGT2*

nomenclature	amino acid exchange	sequence
position 673 fw	R to S	GAACTCGAGAGCGAGATCATCGAGTACATGGCTCGTTTG
position 673 rev		GATGATCTCGCTCTCGAGTTCTTGGAAAGTGTCCATCAA
position 1242/1246 fw	E to D, I to V	ATCCCTAGGGATGAGGTAGAGAAGTGCTTGCTGGAGGCG
position 1242/1246 rev		GCACTTCTCTACCTCATCCCTAGGGATCACCCTGTCTTC

Table S3. Relative concentration (‰ equ. dry weight) of gallic acid, *β*-glucogallin and ellagic acid in different strawberry tissues of *F.* × *ananassa* cv. Calypso and *F. vesca* cv. Yellow Wonder. Levels are calculated in per mil equivalents of the dry weight (‰ equ. dw).

			small green	big green	white	turning	ripe
F. × *ananassa* cv. Calypso	gallic acid	achenes	0.0352 ±0.0007	0.0256 ±0.0012	0.0235 ±0.0004	0.0158 ±0.0014	0.0207 ±0.0003
		receptacles	0.0113 ±0.0013	0.0061 ±0.0018	0.0058 ±0.0007	0.0023 ±0.0006	0.0026 ±0.0002
	β-glucogallin	achenes	0.1043 ±0.0048	0.0853 ±0.0016	0.0864 ±0.0059	0.0776 ±0.0235	0.1036 ±0.0139
		receptacles	0.0285 ±0.002	0.0141 ±0.0013	0.0055 ±0.0002	0.0056 ±0.0006	0.0077 ±0.0008
	ellagic acid	achenes	2.0482 ±0.0627	2.2507 ±0.1238	1.8613 ±0.0257	1.7368 ±0.3322	1.2746 ±0.0588
		receptacles	0.8461 ±0.0873	0.6259 ±0.0853	0.1810 ±0.0035	0.1311 ±0.0038	0.1438 ±0.0086
F. vesca cv. Yellow Wonder	gallic acid	achenes	0.009 ±0.0018		0.0044 ±0.001		0.003 ±0.0007
		receptacles	n.d.		n.d.		n.d.
	β-glucogallin	achenes	0.0228 ±0.0029		0.0111 ±0.0013		0.0115 ±0.0025
		receptacles	0.0035 ±0.0007		n.d.		n.d.
	ellagic acid	achenes	0.2406 ±0.0066		0.1927 ±0.009		0.2288 ±0.0136
		receptacles	0.0499 ±0.0012		0.0349 ±0.0041		0.0333 ±0.0043

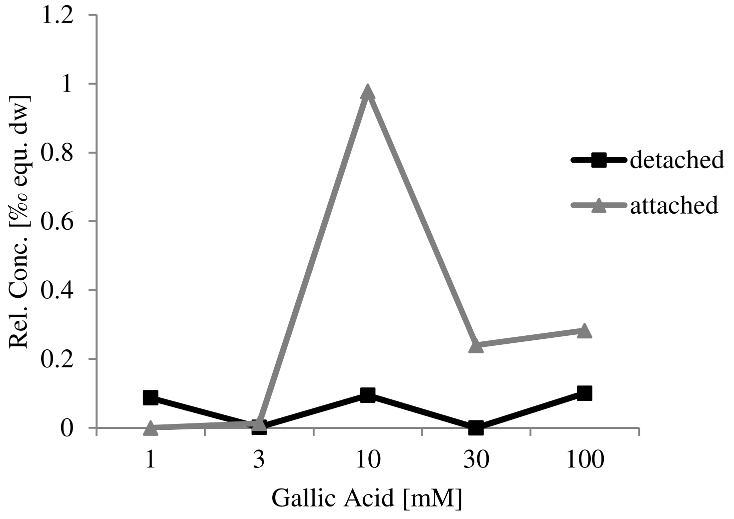

Figure S1. Relative concentration of d_2-β-glucogallin in per mil equivalent of the dry weight, after injection of different amounts of labeled gallic acid (3,4,5-trihydroxybenzoic-2,6-d2 acid; 1-100 mM) into small green strawberry fruits attached to or detached from the plant. Gallic acid levels exceeding 10 mM harm the immature fruits probably due to phytotoxcicity of the phenolic acid.

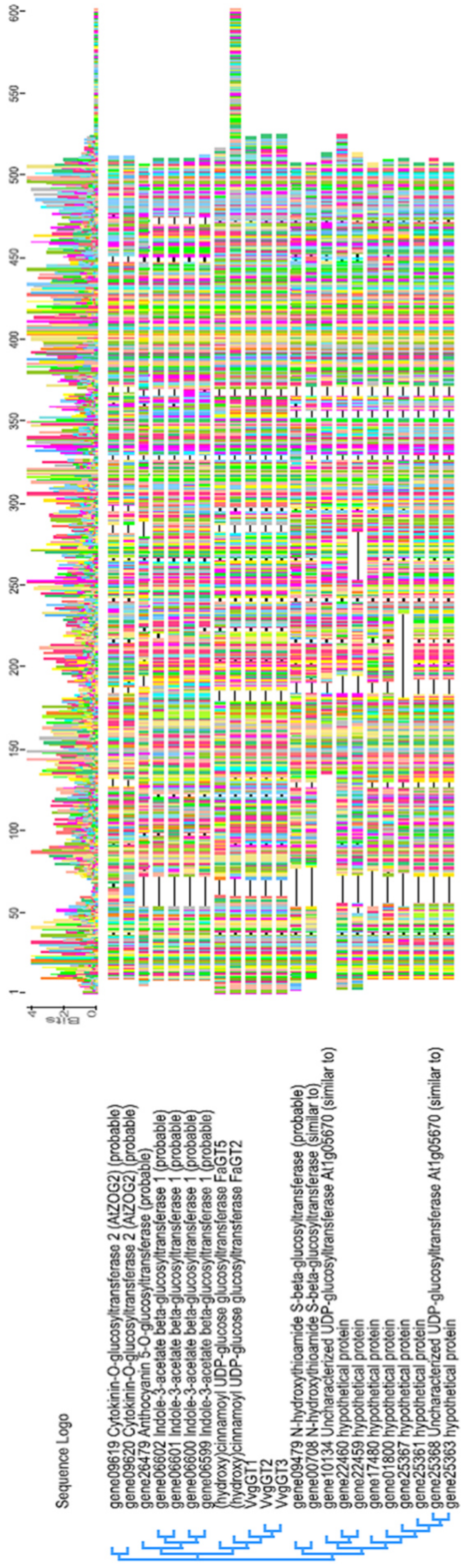

Figure S2. Amino acid sequence alignment (right) and phylogenetic tree (left) of strawberry (*F. vesca*) proteins which show homology to three enzymes from *Vitis vinifera* (VvGT1-3) with gallate glucosyltransferase activity (Khater et al., 2011). Amino acids are represented by a colour code.

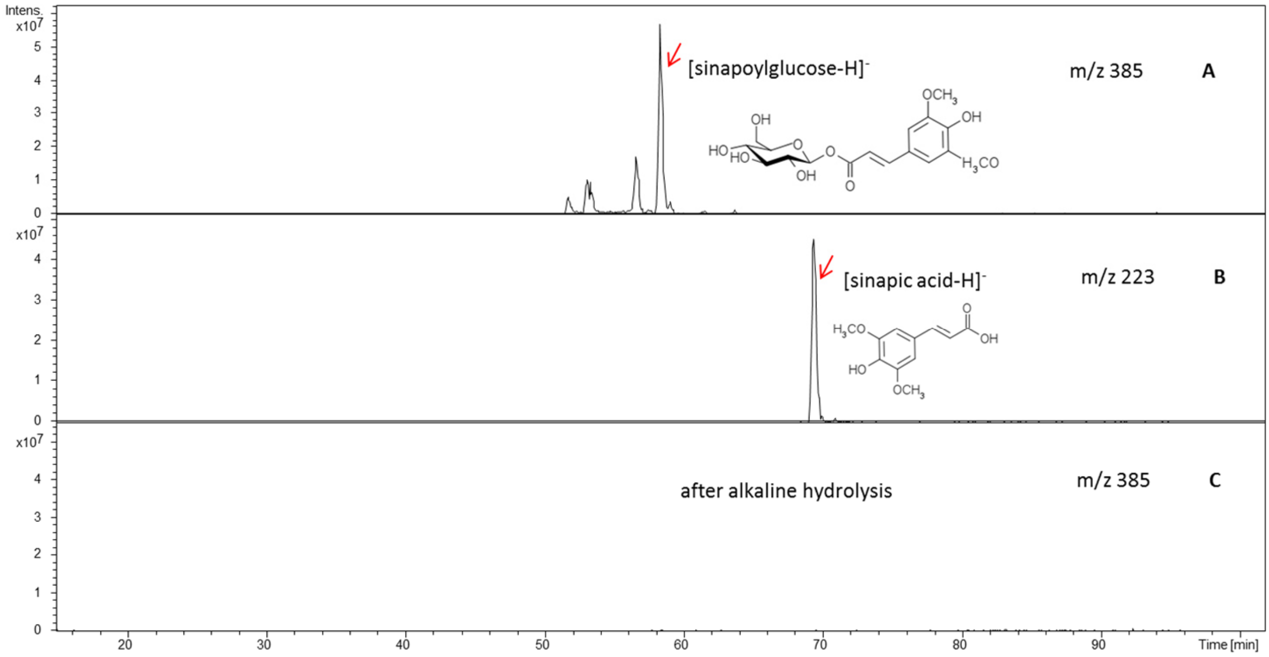

Figure S3. Confirmation of the formation of ester bonds by FaGT2*. Product of FaGT2 before (**A**) and after alkaline hydrolysis (**B**, **C**). After incubation with NaOH (0.3 M, 1 h), sinapoylglucose was hydrolyzed. Signals (A) are probably isomers of sinapoylglucose (Jackson et. al 2001). Sinapic acid (**B**) was released by alkaline hydrolysis.

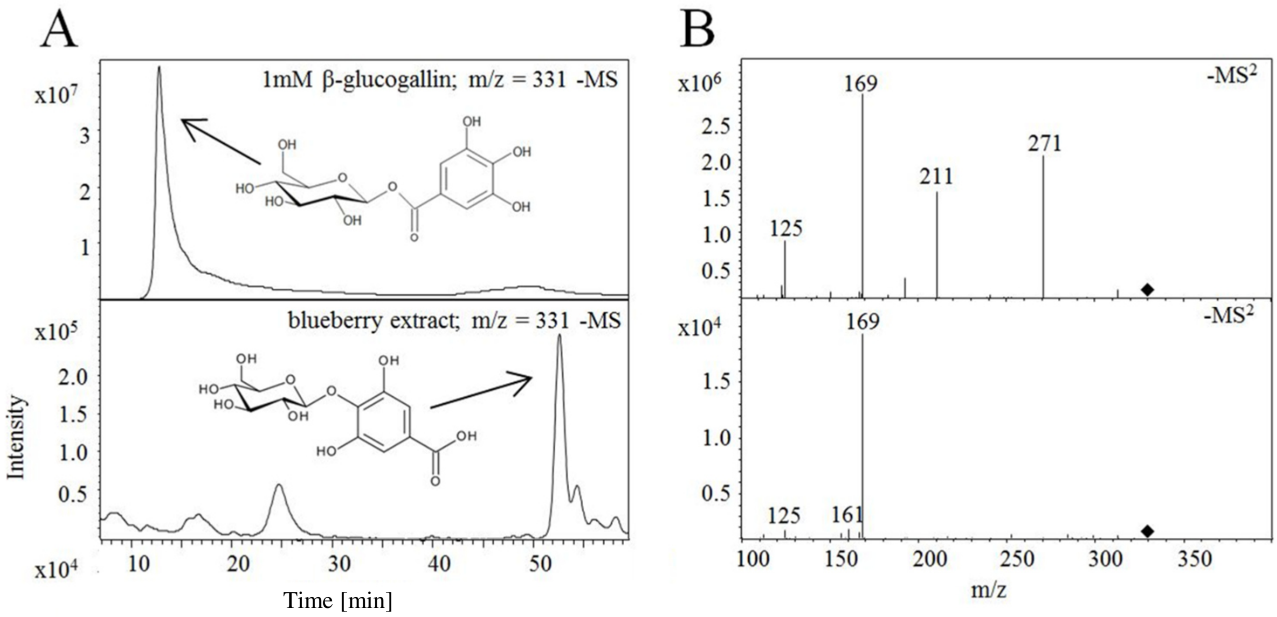

Figure S4. LC-MS extracted ion chromatograms of *m/z* 331 –MS (**A**) and corresponding -MS2 spectra (**B**) of 1 mM *β*-glucogallin authentic chemical and an extract of blueberry containing authentic gallic acid 4-*O*-glucoside (Schuster and Herrmann, 1985).

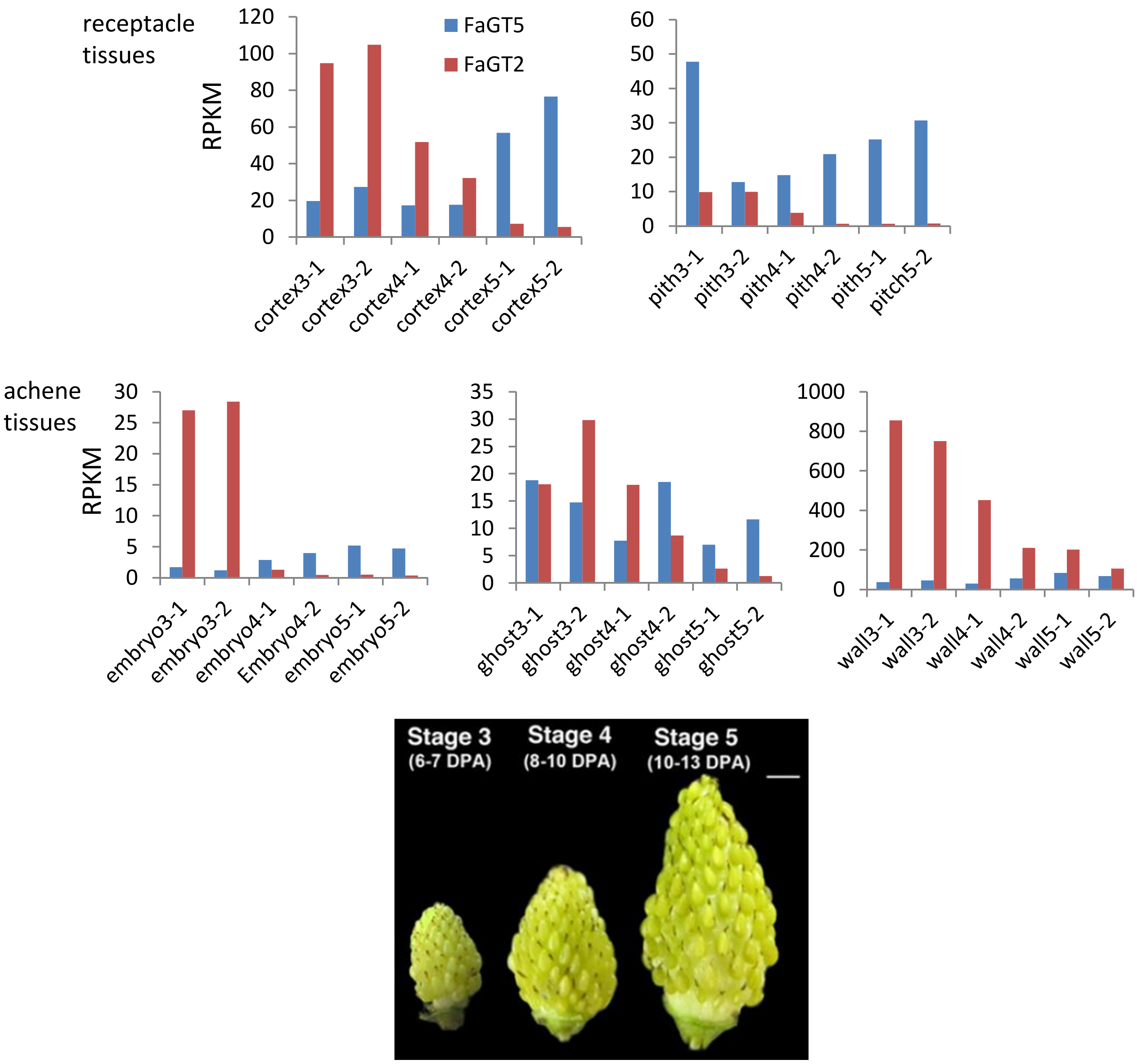

Figure S5. Expression of FaGT2 and FaGT5 corresponding transcripts in three stages of green fruit development of *F. vesca* cv. Yellow Wonder. Top row shows abundance in RPKM in tissues of receptacle (cortex and pith) and second row shows abundance in tissues of achene (embryo, ghost, and wall) in two replicates respectively. Photo shows corresponding stages of fruit development 6-13 days post anthesis. Bars=2mm. Gene expression data and green fruit staging of *F. vesca* development were employed and adapted from SGR database (Kang et al., 2013; Darwish et al., 2013).
